# Predictors of Neonatal Mortality: A Retrospective Cross-Sectional Study From the Special Newborn Care Unit of a Tertiary Care Hospital

**DOI:** 10.7759/cureus.37143

**Published:** 2023-04-04

**Authors:** Sandhya Singh, Roopa Agrawal, Gaurav Agarwal, Abhijit Das, Rupesh Sahu

**Affiliations:** 1 Community Medicine, Government Bundelkhand Medical College, Sagar, Madhya Pradesh, IND; 2 Pediatrics, Government Bundelkhand Medical College, Sagar, Madhya Pradesh, IND; 3 Orthopaedics, Government Bundelkhand Medical College, Sagar, Madhya Pradesh, IND; 4 Community Medicine, Government Chhindwara Institute of Medical Sciences, Chhindwara, IND

**Keywords:** neonatal mortality, special newborn care unit, risk factor, neonatal deaths, predictors

## Abstract

Background

In India, a significant number of newborns die each year, with Madhya Pradesh having the highest neonatal mortality rate. However, there is a lack of information on factors that can predict neonatal mortality.

Objective

This study aimed to examine the factors influencing neonatal mortality among neonates admitted to a tertiary care centre's special newborn care unit (SNCU).

Methods

This retrospective record-based observational study was done at a tertiary care centre, where data from the special newborn care unit (SNCU) from 1st January 2021 to 31st December 2021 was used. We included data of all newborns who were treated in SNCU during the said period and excluded those who got referred or left against medical advice. We abstracted data on age at admission, gender, category, maturity status, birth weight, place of delivery, mode of transport, type of admission, indication of admission, duration of stay and outcome. Qualitative variables were described using frequency and percentage. The chi-square test was used to find out the association of different variables with the outcome, while multivariate logistic regression was conducted to identify risk factors of neonatal mortality. A p-value of <0.05 was considered significant.

Results

We finalized data of 1052 neonates for analysis. Among them, 846 neonates were successfully discharged while 206 neonates were deceased. The major cause of admission was perinatal asphyxia followed by prematurity. The major cause of mortality in this study was sepsis followed by respiratory distress syndrome, birth asphyxia, and prematurity. Mortality of neonates was significantly associated with maturity status, birth weight, place of delivery, age during admission and duration of stay. Prematurity (OR=3.762, 95% CI:1.93-7.33), birth weight 1000-1499g (OR=4.78, 95% CI:2.21-10.32), birth weight <1000g (OR=25.11, 95% CI:5.71-110.24), age at admission <1-day (OR=2.312, 95% CI:1.03-5.19), duration of stay 1-3-days (OR=12.98, 95% CI:7.48-22.52) and <1-day (OR=1271.88, 95% CI:121.39-13325.69) were significant predictors of mortality in our study.

Conclusion

Our study emphasizes monitoring and addressing risk factors like maturity status, birth weight, and age at admission to reduce neonatal mortality, with a focus on early management of preterm births and low birth weight.

## Introduction

The first month after a child’s birth is crucial for survival. During this period, newborns are at their most vulnerable and are at risk of experiencing serious health issues and life-threatening conditions. Unfortunately, despite advances in medical technology and a better understanding of the factors that contribute to neonatal mortality, the number of deaths continues to be alarmingly high in many low- and middle-income countries. Sadly, 2.4 million neonatal deaths occurred worldwide in 2020, despite a global decrease in this statistic. In 2020, 47% of all under-5 deaths occurred in the newborn period. Most neonatal deaths (75%) occur during the first week of life; in 2019, about 1 million newborns died within the first 24 hours [1]. Children who die within the first 28 days of birth suffer from conditions and diseases associated with a lack of quality care at birth or skilled care and treatment immediately after birth and in the first days of life. Preterm birth, childbirth-related complications (birth asphyxia or lack of breathing at birth), infections and birth defects cause most neonatal deaths. Target 3.2 of the Sustainable Development Goals (SDG) aims to end preventable deaths of newborns and children under five years of age by 2030, with a goal for all countries to reduce neonatal mortality to a minimum of 12 deaths per 1000 live births [2]. India contributed the highest number of newborn deaths in 2020 [1]. According to sample registration system data, India’s neonatal mortality rate (NMR) was 20 per thousand live births in 2020 [3]. Moreover, Madhya Pradesh has the highest NMR, accounting for 33 deaths per thousand live births [4]. Though it has been declining at both national and state levels in the last decade, the annual rate of reduction in Madhya Pradesh (0.6%) was lower than that of India (3.8%) [5].

Though studies had been done regarding neonatal mortality and its predictors, most of the studies in this context had been done outside India. There is limited research from India and Madhya Pradesh that gives a comprehensive understanding of neonatal morbidity and mortality data and the factors related to it. So, this study aimed to examine the factors influencing neonatal mortality among neonates admitted to a tertiary care centre’s special newborn care unit (SNCU).

## Materials and methods

Study design, population and settings

We conducted this retrospective record-based observational study in March-June 2022 after getting Institutional Ethical Committee clearance (approval no. IECBMC/67/2022). Data were collected from the medical record department of Government Bundelkhand Medical College, Sagar, Madhya Pradesh. This tertiary care centre serves the population of the Sagar division of Madhya Pradesh. The study population consisted of neonates admitted at this tertiary care hospital’s special newborn care unit (SNCU) from 1st January 2021 to 31st December 2021.

Inclusion and exclusion criteria

We included data of all newborns who were treated in SNCU between 1st January 2021 to 31st December 2021. However, those who got referred or left against medical advice (LAMA) were excluded.

Sample size

A total of 1193 records were found during the study duration. However, 66 neonates were referred and 75 left against medical advice. Therefore, we finalized the data of 1052 neonates as the final sample size.

Data collection tool and study variables

Data were abstracted by the author (second author) herself. Data were abstracted in a data abstraction sheet designed with the help of Epi Info software (version 7.2.4.0, Centers for Disease Control and Prevention, Atlanta, USA). Data abstraction was done for age at admission (age of the neonates at the time of admission; categorized as <1-day/1-6 days/>6-days), gender (male/female), category (general/OBC/SC/ST), maturity status (premature or <37 weeks/full-tern or ≥37 weeks), birth weight (in grams, categorized as <1000g/1000-1500g/1500-2500g/>2500g), place of delivery (private hospital/government hospital/home), mode of transport (personal vehicle/government vehicle), type of admission (indoor/outdoor), indication of admission, duration of stay (<1 day/1-3 days/4-6 days/>6 days) and outcome (survived/expired).

Statistical analysis

IBM Statistical Package for Social Sciences (SPSS) software (IBM Corp., released 2019. IBM SPSS Statistics for Windows, Version 26.0. Armonk, NY: IBM Corp.) was used for data analysis. Qualitative variables were summarized using frequency and percentage. The chi-square test was used to find out the association of different variables with the outcome, while multivariate logistic regression was conducted to identify risk factors of neonatal mortality. A p-value of <0.05 was considered significant.

## Results

Among 1052 neonates, the majority were male children (58.5%), preterm (52.5%), and admitted on the first day of life (65.3%) (Table 1).

**Table 1:** Basic profile of study sample (N=1052) OBC: other backward class; SC: scheduled caste; ST: scheduled tribe

Gender	Frequency	Percentage
Male	615	58.5%
Female	437	41.5%
Category	Frequency	Percentage
General	102	9.7%
OBC	600	57.0%
SC	289	27.5%
ST	61	5.8%
Maturity	Frequency	Percentage
Preterm	552	52.5%
Full term	500	47.5%
Birth weight in grams	Frequency	Percentage
<1000	26	2.5%
1000-1500	115	10.9%
1500-2500	435	41.3%
>2500	476	45.2%
Place of delivery	Frequency	Percentage
Private hospital	165	15.7%
Government hospital	876	83.3%
Home	11	1.0%
Age at admission	Frequency	Percentage
<1 day	687	65.3%
1-6 days	267	25.4%
>6 days	98	9.3%
Mode of transport	Frequency	Percentage
Personal	351	33.4%
Government	701	66.6%
Type of Admission	Frequency	Percentage
Indoor	637	60.6%
Outdoor	415	39.4%
Duration of stay	Frequency	Percentage
<1 day	30	2.9%
1-3 days	395	37.5%
4-6 days	296	28.1%
>6 days	331	31.5%

**Figure 1:**
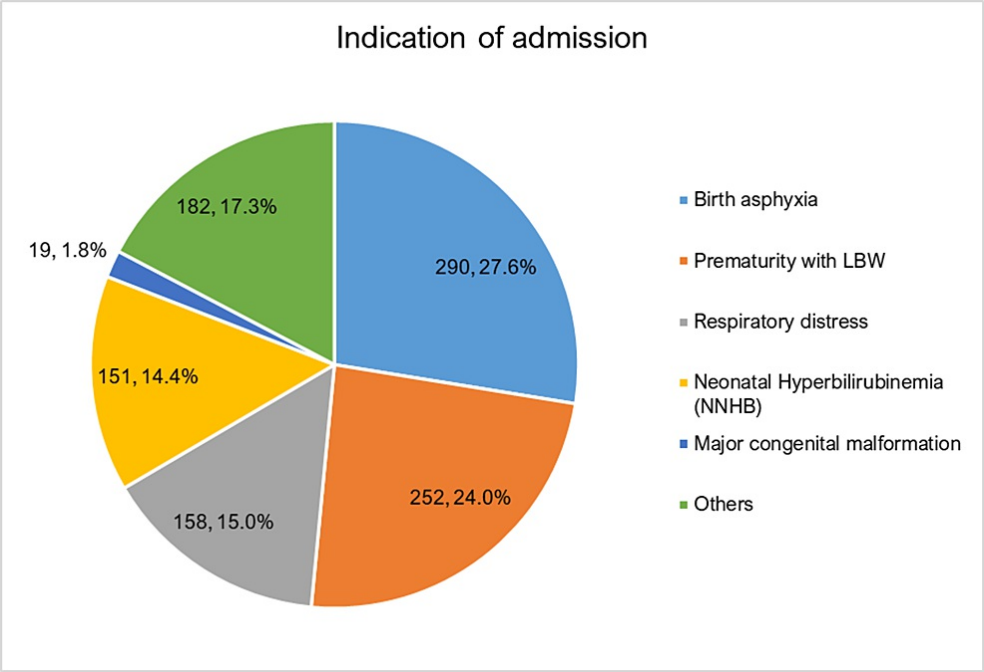
Indication of admission (N=1052) LBW: low birth weight

**Figure 2:**
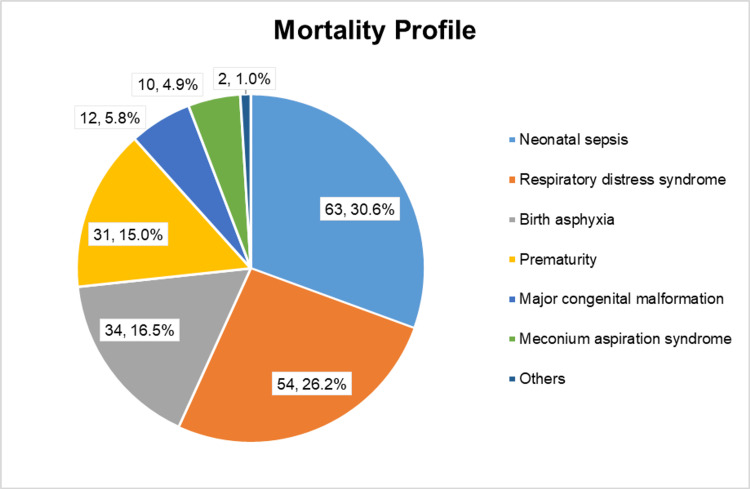
Causes of neonatal mortality (N=206)

Among the 1052 neonates who were admitted to SNCU, 846 neonates were successfully discharged, while 206 neonates were deceased. The major cause of admission was perinatal asphyxia (27.6%), followed by prematurity with low birth weight (23.9%) (Figure 1). While the major cause of mortality in this study was sepsis (30.6%), followed by respiratory distress syndrome (26.2%), birth asphyxia (16.5%) and prematurity (15.1%) (Figure 2).

We applied the chi-square test to determine the association of different variables with neonatal mortality (Table 2). Mortality of neonates was significantly associated with maturity status, birth weight, place of delivery, age during admission and duration of stay. However, we did not find any significant association with gender or type of admission.

**Table 2:** Association of different variables with mortality *Statistically significant

Variables	Subgroup	Survived (%)	Died (%)	Total (%)	P-value
Gender	Female	351 (80.3%)	86 (19.7%)	437 (41.5%)	0.946
	Male	495 (80.5%)	120 (19.5%)	615 (58.5%)	
Category	General	90 (88.2%)	12 (11.8%)	102 (9.7%)	0.131
	OBC	473 (78.8%)	127 (21.2%)	600 (57.0%)	
	SC	236 (81.7%)	53 (18.3%)	289 (27.5%)	
	ST	47 (77.1%)	14 (22.9%)	61 (5.8%)	
Maturity	Full term	443 (88.6%)	57 (11.4%)	500 (47.5%)	<0.0001*
	Preterm	403 (73.0%)	149 (27.0%)	552 (52.5%)	
Birth weight in grams	<1000	3 (11.5%)	23 (88.5%)	26 (2.5%)	<0.0001*
	1000-1500	60 (52.2%)	55 (47.8%)	115 (10.9%)	
	1500-2500	364 (83.7%)	71 (16.3%)	435 (41.3%)	
	>2500	419 (88.0%)	57 (12.0%)	476 (45.2%)	
Place of delivery	Private	122 (73.9%)	43 (26.1%)	165 (15.7%)	0.006*
	Government	718 (82.0%)	158 (18.0%)	876 (83.3%)	
	Home	6 (54.6%)	5 (45.4%)	11 (1.0%)	
Age at admission	<1 day	527 (76.7%)	160 (23.3%)	687 (65.3%)	<0.0001*
	1-6 days	236 (88.4%)	31 (11.6%)	267 (25.4%)	
	>6 days	83 (84.7%)	15 (15.3%)	98 (9.3%)	
Mode of transport	Personal	272 (77.5%)	79 (22.5%)	351 (33.4%)	0.091
	Government	574 (81.9%)	127 (18.1%)	701 (66.6%)	
Type of Admission	Indoor	507 (79.6%)	130 (20.4%)	637 (60.6%)	0.403
	Outdoor	339 (81.7%)	76 (18.3%)	415 (39.4%)	
Duration of stay	<1 day	1 (3.3%)	29 (96.7%)	30 (2.9%)	<0.0001*
	1-3 days	278 (70.4%)	117 (29.6%)	395 (37.5%)	
	4-6 days	266 (89.9%)	30 (10.1%)	296 (28.1%)	
	>6 days	301 (90.9%)	30 (9.1%)	331 (31.5%)	

The output of multivariable logistic regression to predict neonatal mortality is depicted in Table 3. The outcome was taken as the dependent variable in which successfully discharged was coded as “0”, whereas expired was coded as “1”. Logistic regression analysis shows that there was a significant influence of maturity, birth weight, age at admission and duration of stay on mortality of neonates hence these factors are significant predictors of mortality. Whereas gender, category, place of delivery, mode of transport and type of admission had no significant influence on the mortality of neonates admitted to SNCU. The model explained a 47.41% variance in the mortality of neonates and was able to identify 88 cases accurately. The specificity of the model was 98.5% and the sensitivity of the model was 45.1%. Comparing full-term neonates (≥37 weeks) with preterm neonates (<37 weeks), preterm neonates had 3.8 times more mortality. Further on taking into account birth weight in grams, neonates having weight 1500-2499 grams had 1.349 times higher mortality as compared to reference category >2500 grams neonates, 1000-1499 grams neonates had 4.8 times higher mortality as compared to reference category, <1000 grams neonates had 25 times more mortality as compared to the reference category. Considering the duration of stay 4-6 days duration of stay had 1.8 times more mortality as compared to the reference category (>6-days), a 1-3 days duration of stay had approximately 13 times more mortality as compared to the reference category and a <1-day duration of stay had 1271 times more mortality as compared to the reference category. When age at admission was taken into consideration, <1-day neonates had 1.5 times more mortality as compared to the reference category (≥7-days) and 1-6 days neonates had a little less (0.8 times) mortality as compared to the reference category.

**Table 3:** Multivariable logistic regression analysis to predict neonatal mortality * Signifies p-value less than 0.05, ** signifies p-value less than 0.01, *** signifies p-value less than 0.001 OBC: other backward class; SC: scheduled caste; ST: scheduled tribe

	B	S.E.	Wald	OR (95% C.I.)
Gender Male	0.202	0.21	0.932	1.224 (0.81-1.84)
Category	-	-	4.009	-
OBC	0.8	0.427	3.508	2.226 (0.96-5.14)
SC	0.57	0.451	1.596	1.767 (0.73-4.27)
ST	0.779	0.568	1.884	2.18 (0.72-6.63)
Place of delivery	-	-	2.552	-
Government	0.42	0.421	0.995	1.521 (0.66-3.47)
Home	1.318	0.916	2.068	3.735 (0.62-22.50)
Mode of transport Government	0.082	0.273	0.09	1.085 (0.64-1.85)
Type of admission Outdoor	0.656	0.352	3.462	1.926 (0.96-3.84)
Maturity Preterm	1.325	0.34	15.164***	3.762 (1.93-7.33)
Birth Weight (in grams)	-	-	28.416***	-
1500-2499	0.299	0.26	1.329	1.349 (0.81-2.24)
1000-1499	1.566	0.392	15.927***	4.786 (2.21-10.32)
<1000	3.223	0.755	18.229***	25.106 (5.71-110.24)
Duration of stay (in days)	-	-	120.298***	-
4-6	0.595	0.313	3.63	1.814 (0.98-3.34)
1-3	2.564	0.281	83.307***	12.987 (7.48-22.52)
<1	7.148	1.199	35.568***	1271.883 (121.39-13325.69)
Age at admission (in day)	-	-	31.341***	-
<1	0.838	0.413	4.124*	2.312 (1.03-5.19)
1-6	-1.669	0.511	10.689**	0.188 (0.07-0.51)
Constant	-5.813	0.837	48.218***	0.003

## Discussion

This present study was done to find out different predicting factors of neonatal mortality among SNCU-admitted neonates from a tertiary care centre. We found that nearly three-fourths (73.3%) of neonatal deaths were attributed to neonatal sepsis, respiratory distress, birth asphyxia, and prematurity. This finding aligns with the reports from the WHO and the findings of other studies [1,6-9]. This highlights the need for focused interventions to improve neonatal survival, especially during the intrapartum period and the early stages of neonatal life, to decrease neonatal mortality rates.

Our results showed that preterm newborns had nearly four times higher risk of mortality than full term. The maturity status of newborns was a significant predictor of neonatal mortality, which was reported by other studies also [7,10,11]. There are a few potential reasons for this. Some factors may be related to the mother, such as any other health issues she may have had during pregnancy, where she lived, and how often she went for antenatal visits. Other factors may be related to the baby, such as malpresentations, perinatal asphyxia, and Apgar score [12,13]. The study results indicate that there was a statistically significant association between low birth weight and neonatal mortality. Furthermore, the regression model used in the study found that low birth weight was a statistically significant predictor of neonatal mortality. These results align with the findings from similar studies conducted in various countries [6,7,10,11,14,15]. Neonates weighing <1000 grams had 25 times more mortality as compared to the reference category of >2500 grams. However, the wide odds ratio of these has to be considered while interpreting the results. The study highlights the need for interventions aimed at improving birth weight, especially in populations with a high prevalence of low birth weight. This can be achieved through improving antenatal and intranatal care and reducing risk factors such as maternal infections and poor maternal nutrition. Prematures and infants with low birth weight are at a higher risk of developing complications such as hypothermia, infections, and birth asphyxia, which can lead to tissue hypoxia and multi-organ failure. To address these issues and prevent these factors, it is crucial to provide high-quality neonatal care, including effective resuscitation, appropriate thermal support, and adequate feeding. These measures can help to reduce the incidence of these complications and improve the health and survival of preterm and low-birth newborns [10,16,17].

Another factor found to influence neonatal mortality was the duration of hospitalization. Babies who were in the hospital for less than seven days were at a higher risk of death compared to those who stayed for seven or more days. This conclusion aligns with the results of Desalew et al. but contradicts the findings of a study conducted in the Somali region, where a shorter stay in the neonatal intensive care unit (NICU) was associated with a lower mortality rate [7,18]. Though the place of delivery was significantly associated with neonatal mortality, it was not a predictor of mortality in our study. This was in contrast to the study findings of Lee et al. [19]. Opposite to the results of Orsido et al., in our study gender was not a predictor of neonatal mortality [10]. The differences observed may be attributed to variations in the study periods, as well as advancements in the healthcare system. Additionally, there has been a shift in people's attitudes and awareness towards health conditions, leading to an increase in seeking medical attention for neonates.

One of the significant limitations in our research was that only nine factors were considered and other crucial elements such as the method of delivery, number of antenatal care visits, the number of previous pregnancies, urban/rural residency, the mother’s age, socioeconomic status, and the neonate’s Apgar score were not included as data was not available. Furthermore, we did not conduct subgroup, interaction, or sensitivity analyses. Additionally, the single-centre retrospective cross-sectional study design limits the generalizability of our results; further multicentric, prospective studies are recommended.

## Conclusions

Prematurity, low birth weight, place of delivery, duration of stay and age at admission were significantly associated with neonatal mortality in the specialized neonatal care unit. Whereas maturity, birth weight, age at admission and duration of stay were significant predictors of neonatal mortality. Early management of preterm births and low birth weight should be the priority issues for controlling neonatal deaths in institutional settings. The results of this study can be utilized to prioritize and provide appropriate care to newborns who are at high risk and to promote the consistent recording of birth weight and gestational age at birth for better prediction and to ultimately reduce preventable neonatal deaths in settings with limited resources.
